# Impact of Prone Position on 12-Lead Electrocardiogram in Healthy Adults: A Comparison Study with Standard Electrocardiogram

**DOI:** 10.1155/2021/6653061

**Published:** 2021-02-11

**Authors:** Yunis Daralammouri, Murad Azamtta, Hamza Hamayel, Amro Adas, Osama Sawalmeh, Yahia Ismail, Saed H. Zyoud

**Affiliations:** ^1^Department of Cardiology, An-Najah National University Hospital, Nablus, State of Palestine; ^2^Department of Medicine, Faculty of Medicine and Health Sciences, An-Najah National University, Nablus, State of Palestine; ^3^Department of Internal Medicine, An-Najah National University Hospital, Nablus, State of Palestine; ^4^Poison Control and Drug Information Center, Faculty of Medicine and Health Sciences, An-Najah National University, Nablus, State of Palestine; ^5^Department of Clinical and Community Pharmacy, Faculty of Medicine and Health Sciences, An-Najah National University, Nablus, State of Palestine

## Abstract

**Background:**

The standard electrocardiogram (ECG) is commonly performed in the supine posture. It may be difficult to report ECG in a supine posture for those who are unable to adopt the supine posture because of certain circumstances such as acute respiratory distress syndrome—patients who are placed in a prone position for long periods to improve oxygenation. Few data are available on the impact of the prone position on the ECG recording with electrodes on the posterior chest. Examining and analyzing the type and extent of changes observed in the prone ECG in healthy adults have become vitally valuable.

**Methods:**

A cross-sectional observational study enrolled forty healthy adults (24 males and 16 females) aged between 18 and 40 years. The ECG was performed in two different body positions, supine and prone. Influence of prone position on the heart rate, mean QRS axis, amplitude, morphology, duration, mean *T* wave axis and polarity, mean *P* wave axis, PR, and mean QTc duration was evaluated.

**Results:**

The mean heart rate was higher in the prone position (73.2 ± 12.4 bpm) compared with the supine position (69.5 ± 11.5 bpm, *p* = 0.03). The QRS duration decreased considerably from supine (92.8 ± 12.6 ms) to prone (84.9 ± 11.9 ms, *p* < 0.001). The mean QRS axis moved to the left in the prone posture (40.5° ± 32°) relative to the supine (49° ± 28°, *p*=0.015). The QRS amplitude in the precordial leads was significantly decreased from supine (7.42 ± 3.1 mV) to prone (3.68 ± 1.7 mV, *p* < 0.001). In addition, changes in the QRS morphology in leads V1–V3 with the appearance of new *Q* waves were noted. A notable variation in the mean corrected QT (QTc) period with decrease in duration in prone posture ECG (385 ± 64.8) relative to supine (406 ± 18.8, *p*=0.05).

**Conclusions:**

Prone position ECG resulted in significant changes in healthy adults that should be aware of this as this can affect diagnosis and management strategies. Further studies are needed to investigate the impact of prone position on ECG recording in patients with cardiovascular diseases.

## 1. Introduction

The electrocardiogram (ECG) is a widely used tool to study the heart's electrical activity, and it is most frequently used to diagnose different heart conditions. It is an effective method to diagnose ischemic changes, arrhythmia, conduction defects, drug and toxin effects, and electrolyte disturbances [1, 2]. The conventional ECG is usually reported in the supine posture, and the definition of different normal variables is determined on the basis of ECG recordings in the supine position.

However, in those who are unable to adopt the supine posture owing to certain limitations requiring them to take other positions, it may be difficult to report ECG in the supine position. For example, acute respiratory distress syndrome (ARDS) patients are placed in a prone position for long periods during the day to enhance oxygenation and minimize mortality [3–5]. In addition, a prone posture may also be used in morbidly obese patients and patients with burns, skin flaps, or cut wounds in the back of the body.

Moreover, the new evolving coronavirus disease 2019 (COVID-19) infection, which is currently considered a major cause of ARDS, was found to have a significant impact on the heart. Myocardial injury frequency is variable among hospitalized patients with COVID-19, it ranges from 7 to 28%, and it is manifested by decreasing ejection fraction and elevation of troponin I and was an independent risk factor for hospital death [6–8]. These findings make ECG very essential for the evaluation of these patients when they are in the prone position.

Few studies have been conducted to investigate the effect of changing body position on ECG recording. Most of these studies aimed to compare the ECG reported in the supine position with right and left lateral decubitus postures in patients who have been surveyed in intensive care units for myocardial ischemia [9–11]. Other studies have reported the impact of postural changes from supine to setting up and standing position on ECG recording [12–14]. Up to author knowledge, previous studies have compared the effect of position change from supine to prone on ECG recording.

A recently released case report recorded some of the ECG changes observed in the prone position in patients with COVID-19 pneumonia and ARDS [15]. These patients were placed for at least 16 hours during the day in a prone position. While this is highly relevant and important, the type and extent of variation observed in the ECG on the transition in the position from supine to prone in healthy adults must also be examined and quantified. Such modifications will be crucial for the analysis of the ECG performed in the prone position as in patients with ARDS. In these patients, it will also afford perception of the possibility of ECG recording in the prone position.

The current study was conducted to identify and measure ECG changes regarding the mean electrical axis, waveform morphology and amplitude, and different segments and intervals of the position shift from supine to prone position in healthy subjects and to investigate the possibility of ECG recording in routine clinical practice in positions other than supine position.

## 2. Materials and Methods

### 2.1. Study Design, Setting, and Population

This cross-sectional study was conducted between March 1, 2020, and June 30, 2020, at the cardiology department of An-Najah National University Hospital (NNUH), Nablus, Palestine. A convenience sample of 40 healthy nonsmoking males and females aged 18–40 years was randomly selected to be enrolled in this study. Subjects had to meet the following criteria to be included in the study: age 18–40 years, healthy, specifically with no history of previous cardiovascular or respiratory disease, and nonsmoker. Subjects who did not meet the inclusion criteria and those with any concomitant cardiovascular disease were excluded. All participants provided written informed consent for participation in the study. The Institutional Review Board of An-Najah National University approved the study.

### 2.2. Data Collection

Demographic and clinical information were collected from the patients and their medical records. These included age, body mass index, smoking history, and history of any previous medical disease. Vital signs, hemodynamic, and volume status were also assessed. All subjects had echocardiography by cardiologists at An-Najah National University Hospital.

### 2.3. Recording of Electrocardiogram

**Figure 1:**
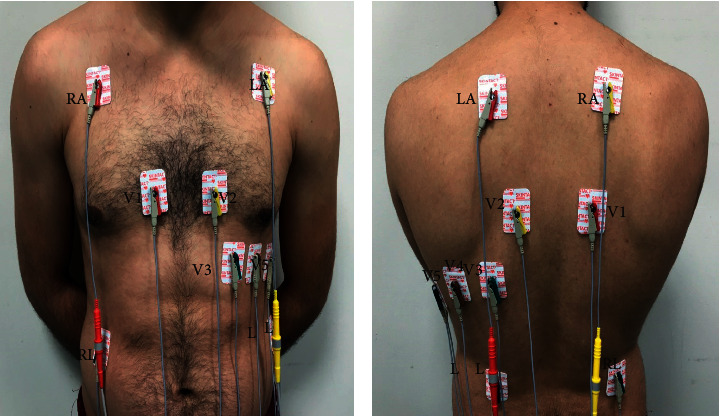
(a) Standard position of anterior precordial leads. The patient's right side is on the left of the picture. (b) Location of precordial leads on posterior chest in prone position. The patient's right side is on the right of the picture. The precordial leads are positioned on the posterior chest opposite to their normal anterior location. The limb leads have preserved their left-right orientation. On the right of the spine is lead V1. Leads V2 through V6 are situated to the left of the spine.

The ECG was recorded in the supine position in accordance with the ECG protocol using a NIHON KOHDEN machine. It was programmed for a paper speed 25 mm/sec and a voltage of 1 mm equals 0.1 mV. Subjects were then placed in the prone position for 15 minutes following which the ECG was recorded with precordial leads placed over the back V1, V2, V4, V3, V5, and V6 as follows; in the right side of the vertebral spine at the 4th intercostal space, at the 4^th^ intercostal space on the left border of vertebral column, in the left 5th intercostal space in midclavicular line, in midpoint between V2 and V4, at left 5th intercostal space in midaxillary line, and in the left 5th intercostal space on the anterior axillary line, respectively (Figure 1).

### 2.4. Data Storage and Analysis

The two forms of ECG were analyzed by 2 cardiologists blinded to the method/posture of the participants. The following variables were examined:

- Heart rate
- Mean QRS vector
- Mean *P* wave vector
- Mean *T* wave vector
- Mean QRS amplitude
- QRS duration
- QRS morphology
- *T* wave polarity
- PR interval
- ST segment changes
- Mean corrected QT interval (QTc)(Bazett's Formula)

All ECG variables were recorded using the computed ECG machine except for the QRS amplitude that was measured to the nearest 0.5 mm by calculating the sum of the highest positive and lowest negative deflection in the limbs and precordial leads with the help of magnifying glass and calipers, and the calibration was 1 mV = 10 mm. ST-T changes reported with positional changes were defined as a deviation (elevation or depression) of at least 1 mm in at least one lead.

### 2.5. Statistical Analysis

The clinical and demographic characteristics of the participants were summarized using descriptive statistics. Means with standard deviation (SD) were used to summarize continuous variables and frequencies with percentages for categorical variables. We used the independent sample t-test and Pearson's chi-square (*χ*^2^) test to examine for any statistically significant differences between characteristics. All outcome variables were normally distributed, and no data transformation was needed. Any *p* value less than or equals 0.05 is considered statistically significant, and all analyses were conducted using SPSS computer software, version 21.0 (IBM Corp).

## 3. Results

Baseline and clinical characteristics of subjects are summarized in Table 1. A total of 40 subjects enrolled in the study, with a mean age of 30.5 ± 6.3 years, ranging from 23 to 39 years, twenty-four (60%) of the subjects were males and the remaining sixteen (40%) were females, and the mean BMI was 25.9 ± 6.3. All subjects were healthy with no previous medical disease.

**Figure 2:**
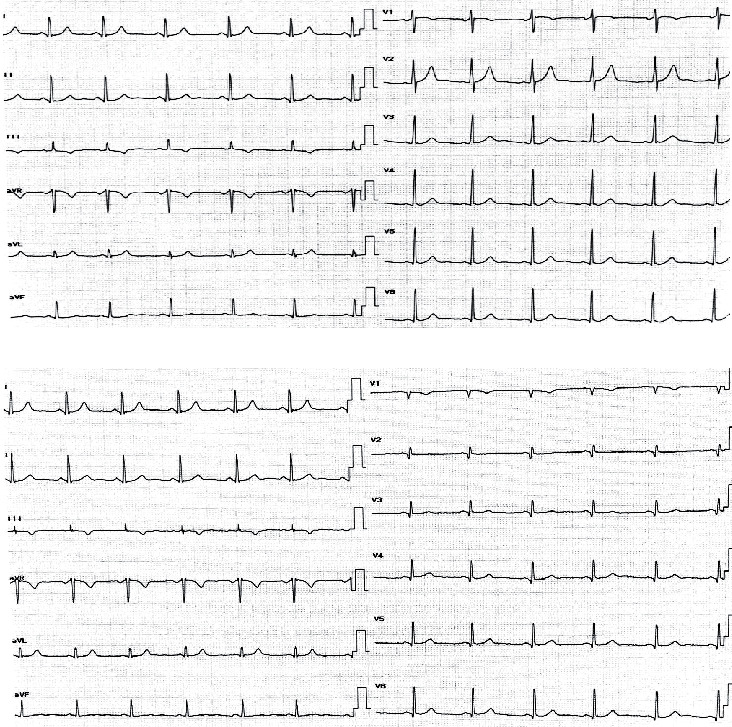
(a) Supine ECG, normal sinus rhythm, and normal frontal QRS axis. (b) Prone ECG in the same patient. Low QRS amplitude was noted in precordial leads, and new *Q* waves were seen in leads V1 to V3.

Figure 2 shows two ECGs performed in the supine and prone position. The heart rate has markedly increased in the prone position (73.2 ± 12.4 bpm) compared with the supine position (69.5 ± 11.5 bpm, *p*=0.03). In addition, there was a notable alteration in the mean QRS axis between the supine and prone ECG with a shift of the mean QRS axis to the left in the prone position (40.5° ± 32°) compared with the supine position (49° ± 28°, *p*=0.015). However, no significant variations were noted in the mean *P* wave axis of postural changes from supine (43.4 ± 22.6) to prone (47.6 ± 27.3, *p*=0.36) as well as the mean *T* wave axis in supine (43.6 ± 20.3) and prone (39.1 ± 20.6, *p*=0.86). The QRS amplitude in the precordial leads had a substantial reduction with the change of position from supine (7.42 ± 3.1 mV) to prone (3.68 ± 1.7 mV, *p* < 0.001), while there were no significant changes in the QRS amplitude in the limb lead (*p*=0.513) (Table 2) and (Figure 2).

The QRS duration has noticeably decreased with the position shift from supine (92.8 ± 12.6 ms) to prone (84.9 ± 11.9 ms, *p* < 0.001). Furthermore, the QTc interval is calculated using the Bazett formula; QTc (ms) = QT measured/[square root of (RR)] (where RR is the RR interval) has significantly reduced with the change of position from supine (406 ± 18.8) to prone (385 ± 64.8, *p*=0.05). However, there was no considerable variation in the PR interval with change position (155.6 ± 24.8 ms) from supine to prone (159.1 ± 27.2 ms, *p*=0.37) (Table 2) and (Figure 2).

Prone ECG showed important variations in the QRS morphology in precordial leads V1 and V2. While RS was the main morphology in the supine position, QR was the predominant morphology in the prone position (Table 3) and (Figure 2). Moreover, the *T* wave polarity in the precordial leads V1 and V2 has changed with the shift of position from supine to prone. It was negative in 70% and 95% of cases in V1 and V2, respectively, in the supine position, while it was negative in 95% and 47.5% of cases in V1 and V2, respectively, in the prone position (Table 3) and (Figure 2). On the other hand, no considerable variations in the ST segment were noted in the prone position ECG.

## 4. Discussion

It is widely agreed that postural changes of the body can have a significant effects on the ECG recording, particularly on the electrical axis of the heart, wave amplitude, and ST segment. These changes were linked to variations in the heart position inside the thorax as a result of postural changes in addition to changes in lung volume [16, 17]. There have been several studies in the past evaluating the impact of postural changes (supine, sitting, standing, and lying) on the ECG, but no previous studies have examined the effect of prone position on ECG recording.

In our study, ECG has been recorded in two distinct positions, supine and prone, in 40 healthy adults. Twenty-four (60%) of the subjects were males and 16 (40%) were females; the mean age was 30.5 ± 6.3 years. Influence of prone position on the heart rate, mean QRS axis, amplitude and duration, *P* wave axis, *T* wave axis and morphology, PR, and QTc duration was evaluated, and the association between these ECG variables and postural changes of the body were analyzed.

In this analysis, the heart rate has significantly increased in the prone position (73.2 ± 12.4 bpm) compared with the supine position (69.5 ± 11.5 bpm, *p*=0.03). This result was also supported by several earlier studies that demonstrated a considerably higher heart rate in the prone posture [18, 19]. This fact must be addressed when ECG is performed in a prone position for patients with sepsis and hypovolemia as the heart rate plays an important role in determining the response of these patients to various forms of care. The finding of a higher heart rate in prone posture was explained by reduction in venous preload (inferior vena cava compression) and a rise in resistance to left ventricular filling (due to increased intrathoracic pressure), leading to a lower stroke volume [20]. This was assumed to lower arterial pulse waves that inhibited baroreflexes and consequently enhanced nervous sympathetic activity and increased the heart rate [19].

In comparison with the supine position, the prone ECGs revealed significant changes in the mean QRS axis and suggest that such observations can contribute to erroneous interpretations. It was noted that the QRS axis was shifted to left in the prone position (40.5° ± 32°) compared with the supine position (49° ± 28°, *p*=0.015). Therefore, in the presence of these changes, we cannot rely on the prone position ECG to detect left axis deviation and left anterior fascicular block. This is in accordance with previous studies that documented this axis change. These shifts were contributed to variations in the anatomical location of the heart inside the thorax. Whereas a relatively hard tissue holds the heart at its base, the apical end of the heart can move significantly inside the chest with simple movement of patients, leading to changes in the electric vectors [21, 22]. In our study, in addition to the change in body position, the electrode location was also changed from the anterior chest wall to the posterior chest.

A comparison of the mean QRS amplitude between the prone and standard supine ECG revealed a significant decrease in the precordial lead in the prone position (3.68 ± 1.7 mV) compared with supine position (7.42 ± 3.1 mV, *p* < 0.001), while there was no difference in the limb leads. This would affect the conventional criteria used for LVH diagnosis; minimal ST segment changes in precordial leads can also be significant. When patients are in a prone posture, their heart drops ventrally and shifts caudally along the anterior chest wall [22]. Looking from the back, the heart is further away from the posterior chest lead. The further distal location of the heart combined with greater impedance from soft tissue (lungs and mediastinum) and bones (scapula and vertebrae) between the displaced myocardium and the electrodes can explain our finding of a smaller QRS amplitude in precordial leads; the condition may be aggravated by diseased lungs.

**Figure 3:**
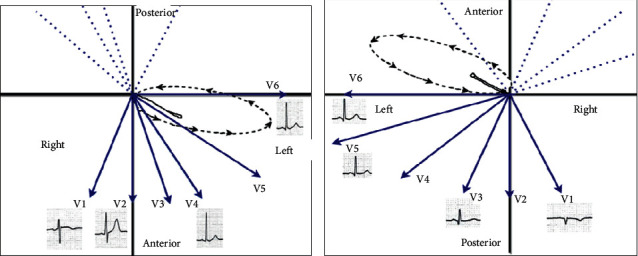
The correlation between QRS vector loops and ECG tracing has been explained. From the centre of the x-y-z axes, QRS waveform circuits are initiated. Sequential 2-ms increments during ventricular depolarization are marked by arrows. On the ECG leads tracing, the amplitude of QRS complexes is consistent with the vertical direction of the vector loop at a specific location and time. (a) In the supine position, the horizontal vector loop showed that, at first, the ventricular depolarization loop was anteriorly and leftward directed towards the anterior chest precordial leads (V1 to V3), resulting in initial positive deflection in these leads. (b) In the prone position, the horizontal vector loop showed that the ventricular depolarization loop was initially oriented anteriorly and leftward away from precordial leads V1 to V3, which are positioned on the back of the patient, resulting in new *Q* wave emergence and low QRS amplitude.

When ECGs were recorded for patients in prone posture, precordial leads were always shown to have comparatively smaller *P* waves and notable *Q* waves in leads V1 through V3, which contributed to the misinterpretation as an anteroseptal myocardial infarction. These variations can be clarified through the vectorcardiograms [23, 24]. The vector loop tracks the course of the action potential during the cardiac cycle. For example, the ventricle stimulation initially progresses from the left bundle to the interventricular septum and then to the left and right ventricular walls. The interaction between the vector loops and the limb leads on the ECG tracing does not significantly change between the supine and the prone posture in the frontal plane. However, the vector loops are shifted anteriorly far from the posterior lead in the horizontal plane, clarifying the emergence of *Q* waves in the prone position on the surface ECG (Figure 3).

Prone ECGs have demonstrated important differences in QRS duration to change position from supine to prone, interestingly; QRS duration was shorter in the prone position (84.9 ± 11.9 ms) compared with the supine position (92.8 ± 12.6 ms, *p* < 0.001). This statement should be considered when analyzing prone ECG looking for bundle branch blocks, pre-excitation syndrome, and ventricular arrhythmias. These differences in the QRS interval can be explained by the fact that QRS duration is correlated with the heart size [25]. Previous studies have shown that both diastolic and systolic volumes change in response to postural variations [26]. Owing to the decrease of venous blood flow (caused by compression of the inferior vena cava), in addition to compression of the heart within the thoracic cavity, the heart size will be smaller in the prone position.

The current study also revealed changes in the mean QTc interval with the change of position from supine (406 ± 18.8) to prone (385 ± 64.8, *p*=0.055). This observation has to be taken into account when interpreting prone ECG recordings, as it will lead to spurious interpretation when investigating arrhythmogenic QT syndromes, drug side effects, and electrolyte abnormalities. As stated earlier, the prone posture is associated with a significantly shorter QRS duration, which also contributes to the shortening of the QT interval. Differences in the QTc interval with postural changes have been confirmed in many previous studies, emphasizing that position changes can result in variations in cardiac repolarization [27–29].

Our study has several limitations, including the small sample size and all subjects involved in this study are healthy and have no cardiorespiratory disease. Further studies in these patients are required to compare the pathological findings of ECG in prone and supine positions.

## 5. Conclusions

A shift of body position from supine to prone with displacing precordial leads on the posterior chest causes changes in the ECG recording. These changes include variations in the heart rate, mean QRS axis, amplitude, duration, and morphology, as well as changes in the mean corrected QT interval (Bazett's formula). These variations need to be considered when ECG is performed in a prone position because of medical reasons, such as patients with COVID-19 and ARDS.

## Figures and Tables

**Table 1:** Baseline demographic and clinical characteristics of patients.

Variable	Mean ± SD or frequency (%)
Age^*∗*^	30.5 ± 6.3

*Gender, n (%)* ^*∗∗*^	
Female Male	16 (40%) 24 (60%)

*Smoking, n (%)* ^*∗∗*^	
No Yes	40 (100%) 0 (0%)

*Medical Disease, n (%)* ^*∗∗*^	
No Yes	40 (100%) 0 (0%)

Body mass index^*∗*^	25.9 **±** 6.3

^*∗*^Statistical significance of differences calculated using the independent sample *t*-test. ^*∗∗*^Statistical significance of differences calculated using the Pearson's chi-square (*χ*^2^) test.

**Table 2:** Effect of prone position on heart rate, mean electrical axis, QRS amplitude, QRS duration, PR, and QTc interval.

Variable	Supine	Prone	*P* value^*∗*^
Heart rate (bpm)	69.5 ± 11.5	73.2 ± 12.4	0.03
Mean QRS vector (degree)	49.8 ± 28	40.5 ± 32	0.015
Mean *T* wave vector (degree)	43.6 ± 20.3	39.1 ± 20.6	0.86
Mean *P* wave vector (degree)	43.4 ± 22.6	47.6 ± 27.3	0.36
Mean QRS amplitude in the precordial leads (mV)	7.42 ± 3.1 mV	3.68 ± 1.7 mV	<0.001
Mean QRS amplitude in limb leads (mV)	4.74 ± 0.86 mV	4.68 ± 1.1 mV	0.513
QRS duration	92.8 ± 12.6 ms	84.9 ± 11.9 ms	<0.001
PR interval	155.6 ± 24.8 ms	159.1 ± 27.2 ms	0.37
Mean QTc (ms)	406 ± 18.8	385 ± 64.8	0.05

QTc: Corrected QT interval. Data are expressed as mean ± SE. ^*∗*^Statistical significance of differences calculated using the independent sample *t*-test.

**Table 3:** Effect of prone position on QRS morphology and *T* wave polarity in precordial leads.

Precordial leads		Supine position ECG	Prone position ECG
*V1*	QRS morphology	RS in 77.5% of cases	QR in 72.5% of cases
	*T* wave polarity	Negative in 70% of cases	Negative in 95% cases

*V2*	QRS morphology	RS in 70% of cases	QR in 90% of cases
	*T* wave polarity	Positive in 95% of cases	Negative in 47.5% cases

*V3*	QRS morphology	RS in 52% of cases	QR in 100 of cases
	*T* wave polarity	Positive in 95% of cases	Negative in 95% of cases

Data are expressed as percentages. ^*∗∗*^Statistical significance of differences calculated using the Pearson's chi-square (*χ*^2^) test.

## Data Availability

All data are included within the article. The raw data are available from the corresponding author upon request.

## Abbreviations

ECG: Electrocardiogram
ARDS: Acute respiratory distress syndrome
COVID-19: Coronavirus disease 2019
NNUH: Najah National University Hospital
SPSS: Statistical Package for the Social Sciences.
